# Crowdsourced Feedback to Improve Resident Physician Error Disclosure Skills: A Randomized Clinical Trial

**DOI:** 10.1001/jamanetworkopen.2024.25923

**Published:** 2024-08-07

**Authors:** Andrew A. White, Ann M. King, Angelo E. D’Addario, Karen Berg Brigham, Joel M. Bradley, Thomas H. Gallagher, Kathleen M. Mazor

**Affiliations:** 1 Department of Medicine, University of Washington School of Medicine, Seattle; 2 National Board of Medical Examiners, Philadelphia, Pennsylvania; 3 Collaborative for Accountability and Improvement, University of Washington School of Medicine, Seattle; 4 Department of Medicine, Geisel School of Medicine at Dartmouth, Hanover, New Hampshire; 5 Department of Pediatrics, Geisel School of Medicine at Dartmouth, Hanover, New Hampshire; 6 Department of Bioethics, University of Washington School of Medicine, Seattle; 7 Department of Medicine, UMass Chan Medical School, Worcester, Massachusetts

## Abstract

**Importance:**

Residents must prepare for effective communication with patients after medical errors. The video-based communication assessment (VCA) is software that plays video of a patient scenario, asks the physician to record what they would say, engages crowdsourced laypeople to rate audio recordings of physician responses, and presents feedback to physicians.

**Objective:**

To evaluate the effectiveness of VCA feedback in resident error disclosure skill training.

**Design, Setting, and Participants:**

This single-blinded, randomized clinical trial was conducted from July 2022 to May 2023 at 7 US internal medicine and family medicine residencies (10 total sites). Participants were second-year residents attending required teaching conferences. Data analysis was performed from July to December 2023.

**Intervention:**

Residents completed 2 VCA cases at time 1 and were randomized to the intervention, an individual feedback report provided in the VCA application after 2 weeks, or to control, in which feedback was not provided until after time 2. Residents completed 2 additional VCA cases after 4 weeks (time 2).

**Main Outcomes and Measures:**

Panels of crowdsourced laypeople rated recordings of residents disclosing simulated medical errors to create scores on a 5-point scale. Reports included learning points derived from layperson comments. Mean time 2 ratings were compared to test the hypothesis that residents who had access to feedback on their time 1 performance would score higher at time 2 than those without feedback access. Residents were surveyed about demographic characteristics, disclosure experience, and feedback use. The intervention’s effect was examined using analysis of covariance.

**Results:**

A total of 146 residents (87 [60.0%] aged 25-29 years; 60 female [41.0%]) completed the time 1 VCA, and 103 (70.5%) completed the time 2 VCA (53 randomized to intervention and 50 randomized to control); of those, 28 (54.9%) reported reviewing their feedback. Analysis of covariance found a significant main effect of feedback between intervention and control groups at time 2 (mean [SD] score, 3.26 [0.45] vs 3.14 [0.39]; difference, 0.12; 95% CI, 0.08-0.48; *P* = .01). In post hoc comparisons restricted to residents without prior disclosure experience, intervention residents scored higher than those in the control group at time 2 (mean [SD] score, 3.33 [0.43] vs 3.09 [0.44]; difference, 0.24; 95% CI, 0.01-0.48; *P* = .007). Worse performance at time 1 was associated with increased likelihood of dropping out before time 2 (odds ratio, 2.89; 95% CI, 1.06-7.84; *P* = .04).

**Conclusions and Relevance:**

In this randomized clinical trial, self-directed review of crowdsourced feedback was associated with higher ratings of internal medicine and family medicine residents’ error disclosure skill, particularly for those without real-life error disclosure experience, suggesting that such feedback may be an effective way for residency programs to address their requirement to prepare trainees for communicating with patients after medical harm.

**Trial Registration:**

ClinicalTrials.gov Identifier: NCT06234085

## Key Points

**Question:**

Is crowdsourced feedback from laypeople an effective educational intervention to improve resident physicians’ error disclosure communication skills?

**Findings:**

In this randomized clinical trial including 146 second-year internal medicine and family medicine residents, practice with simulation software followed by feedback from crowdsourced laypeople was associated with a modest increase in communication ratings on a 5-point scale among residents who reviewed their feedback.

**Meaning:**

The findings from this trial suggest that self-directed review of crowdsourced feedback is an effective way for residency programs to address their requirement to prepare trainees for communicating with patients after medical harm.

## Teaser

This randomized clinical trial examines the effectiveness of video-based communication assessment feedback in preparing resident trainees to communicate with patients after errors resulting in medical harm.

## Introduction

Following harmful medical errors, physicians often feel ill-equipped to communicate with patients and families.^1,2,3,4^ Incomplete or poor physician communication magnifies the pain and uncertainty experienced by patients and impairs efforts to improve patient safety.^5,6^ To better prepare physicians, the Accreditation Council for Graduate Medical Education requires that all residents receive training and practice in adverse event disclosure to patients.^7^ However, 23% of US residencies provided no such training in 2021.^8^ Most other programs provided only informal training or lectures, approaches that are necessary but likely insufficient. Lectures do not ensure communication skill acquisition, and informal training falls short because real-life disclosure is unpredictable and often concludes without formative feedback from supervisors or harmed patients.^9,10,11,12^ To supplement lectures and bedside learning, educators need practical tools for residents to practice simulated medical error disclosure and receive reliable, patient-centered formative feedback. The video-based communication assessment (VCA) is software for this purpose, but limited evidence exists regarding its effectiveness.

The VCA provides physicians with practice and feedback on their communication skills.^13^ It presents videos of vignettes and prompts users to audio-record what they would say to the patient. Recorded responses are rated by web-based panels of laypeople responding as if they were the patient in the scenario.^14^ The laypeople are recruited via Amazon Mechanical Turk (MTurk), a crowdsourcing website with a large and diverse participant population.^15,16^ Physicians receive feedback reports with summary ratings of their performance, average peer scores, learning points derived from raters’ comments, and audio of highly rated peer responses. VCA feedback reports are designed to support self-directed communication skill learning through multiple aspects of deliberate practice.^17,18,19^ First, learning points reinforce desired behaviors and help learners to reconstruct task knowledge around the approach desired by patients. Second, listening to exemplars aids the conceptualization of ideal performance on specific communication subtasks. For example, cases are organized around challenging questions raised by patients that physicians may struggle to address without training and practice^1^ (eg, “Why did this happen?” or “Who is going to pay for this care?”). Third, personal ratings help learners to gauge relative performance and determine areas for further practice.

In prior studies,^20,21^ the VCA proved highly acceptable and feasible for preparing learners for common communication scenarios, and raters generated high-quality, actionable feedback. For VCA cases presenting harmful medical errors, panels of crowdsourced laypeople provided ratings that were consistent with those of patients with personal experience with harmful error.^22^ In a single-site pre-post pilot study involving paid resident volunteers from 3 specialties, standalone VCA practice without a didactic curriculum was associated with an increase in ratings of residents’ error disclosure skills.^23^ Because the effectiveness of the VCA has not been assessed, we sought to test the effect of formative feedback delivered by VCA with a large multisite cohort as part of an error disclosure curriculum. This article describes a randomized clinical trial to test the hypothesis that residents’ error disclosure skills, as assessed by laypeople, would improve after reviewing reports with personal performance feedback and recommendations for effective error disclosure.

## Methods

From July 2022 through May 2023, we conducted a single-blinded, multicenter, randomized clinical trial of the effect of crowdsourced ratings and feedback on postgraduate year 2 (PGY2) internal medicine (IM) and family medicine (FM) resident physicians’ medical error communication skills (see the trial protocol in Supplement 1). The University of Washington institutional review board ruled this study exempt from review. Participants were not compensated. No VCA results were shared with residency faculty. Risks and benefits were explained verbally; participation was considered to indicate consent. Residents could participate in the training and opt out of research. This report follows the Consolidated Standards of Reporting Trials (CONSORT) reporting guideline for randomized studies.^24^

### Setting and Participants

Participants attended IM and FM residencies at 7 US academic medical centers: University of Washington, Seattle (IM and FM); University of Washington, Boise (IM); Washington State University, Everett (IM); Beaumont University (IM at Dearborn and Royal Oak, FM at Wayne and Troy); Dartmouth-Hitchcock Medical Center (IM); University of Massachusetts, Worcester (IM); and Washington University, St. Louis (IM). Each residency participated during a 4- to 8-week window chosen by program leaders to optimize PGY2 residents’ availability. Before the study, none of the residencies provided programwide required error disclosure training. We chose IM and FM residencies because of their large size and shared familiarity with medical cases involving adults. We enrolled only PGY2 residents to control for years of training and simplify scheduling. Residents were eligible for the study if they were on any clinical or nonclinical rotation that provided protected time to attend the teaching conference chosen by their program for VCA practice. Residents were not eligible if they were on leave at the time of the study.

### Error Disclosure Training

Programs assigned all eligible PGY2 residents to attend a 75-minute teaching session at time 1, consisting of 50 minutes of lecture about communication with patients after medical errors, 20 minutes of VCA practice with 2 cases (containing 4 and 3 sequenced vignettes, respectively), and 5 minutes of debrief. At time 2, residents attended a session consisting of 25 minutes of lecture about institutional programs to support clinicians with error disclosure and 20 minutes of VCA practice with 2 additional cases (3 sequenced vignettes each). The recommended duration between time 1 and time 2 was 4 weeks, although the conference schedule at 2 residencies required an interval of 5 to 8 weeks for some residents. The training took place during regularly scheduled conferences for PGY2 residents. The lectures were delivered over video conference by investigators experienced with communication skills training (A.A.W. and T.H.G.). The lecture was adapted from published curricula and modified to highlight site-specific event review policies and clinician support systems.^25,26^ Residents were encouraged to complete the VCA during the allocated conference time, but could complete it within 5 days if necessary. The study ended when all teaching conferences organized by programs had concluded.

### Intervention

**Figure 1.**
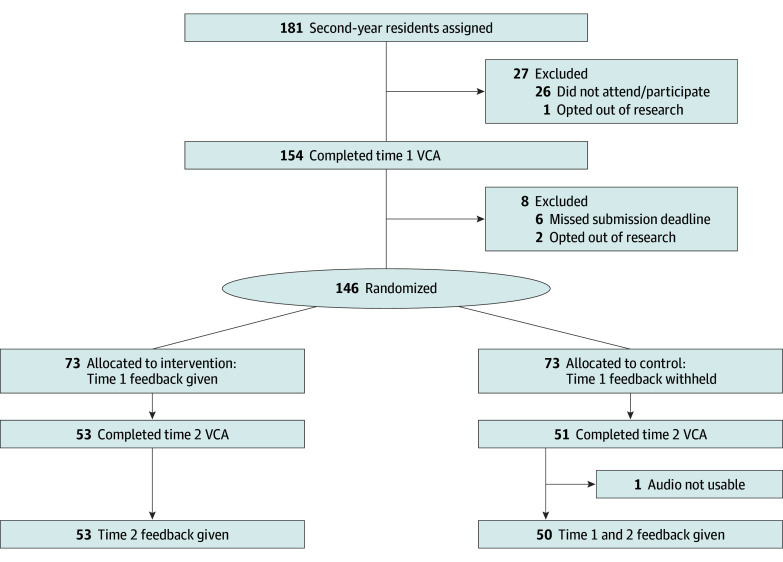
Participant Enrollment Flowchart VCA indicates video-based communication assessment.

**Figure 2.**
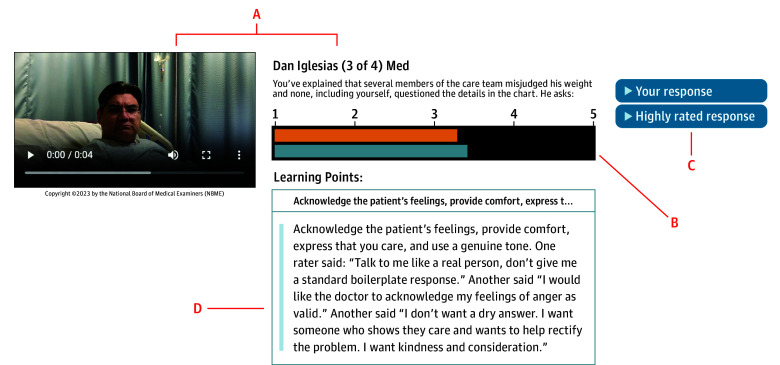
Video-Based Communication Assessment Feedback Interface for 1 Vignette Within an Error Disclosure Case Video-based communication assessment feedback components include the case text and video prompt available for review (A), personal overall rating from the panel of crowdsourced raters (orange) and peer average (blue) (B), buttons that play the recorded response to this vignette and an exemplar response from a highly rated peer (C), and learning points derived from crowdsourced advice about what they would like the physician to say in this situation (D). Reprinted with permission from the National Board of Medical Examiners.

Residents who completed the VCA at time 1 were randomized in 1:1 fashion to either receive feedback before time 2 (intervention) or after time 2 (control) (Figure 1). Block randomization was performed centrally in variable block sizes, before time 1 responses were scored, by a coinvestigator (A.E.D.) with access to lists of the nonidentifying coded usernames of residents who completed time 1. Investigators and raters were blinded to assignments. Residents were unblinded after feedback was released. Intervention residents received automated emails when their feedback was available, instructing them to review it in the application (app) before the next teaching session and VCA practice. Feedback was typically provided 2 weeks after VCA use to allow for completion of rating and data quality checks. Reports presented an interactive feedback display within the VCA app for each vignette (Figure 2). We asked residents receiving the intervention not to discuss feedback with colleagues to avoid contamination.

### VCA App and Cases

The VCA app used in this study has been described previously.^13,18^ Users entered the app with a personal login and password to access vignettes or review feedback. This study used 4 cases, including 2 previously described cases (a delayed diagnosis of breast cancer and an anticoagulant overdose).^20,21^ We created 2 new cases depicting a delayed diagnosis of sepsis and the development of a pressure sore (eTable 1 in Supplement 2). The cases were tested and refined with feedback from 6 faculty members in IM or FM to improve relevance, clarity, and believability. We designed all cases to reflect serious safety events of equivalent preventability and harm severity. Professional actors portrayed each patient or family member.

### Audio Collection and Rating

Residents provided audio responses to each vignette through the VCA software. Audio responses were bundled into rating tasks on MTurk for raters who were US residents aged 18 years or older and able to speak and read English. Raters answered demographic questions, read a vignette description in lay language, viewed the patient video, and listened to resident responses. They rated each response on 6 items covering domains related to accountability, honesty, apology, empathy, caring, and overall response, using a previously described instrument.^21^ Items used a 5-point scale anchored with the labels poor, fair, good, very good, and excellent. After rating a set of responses, the rater responded in free text to the question, “What would you want the provider to say if you were the patient in this situation?” A power analysis based on previous research^23^ with a moderate η_p_^2^ of 0.09, determined that a sample of 96 PGY2 residents was needed to achieve a power of 0.85 at α = .05 for the analysis of covariance (ANCOVA) to effectively test the study hypotheses.

We sought at least 6 raters per response after removing raters with indications of low contributions to reliability.^27^ To eliminate inattentive raters from quantitative analysis, open-ended responses were analyzed for quality. One analyst reviewed all responses and flagged responses that bypassed the question (eg, none, good, or NA [not applicable]), were generic, repetitive for multiple vignettes, or were copied and pasted from the ratings task questions (eg, “the provider understood how I was feeling”). A second analyst reviewed and confirmed all exclusions.

### Resident Surveys

Residents completed questionnaires in the VCA application before proceeding to cases. The survey at time 1 asked about age, gender, race, the number of times the resident had personally participated in disclosure of a harmful error to a patient or family, and their highest level of involvement during disclosure of a harmful medical error. Data on race were included in this study because this information would be valuable for future analyses to address racial concordance between users and raters. Before time 2, residents who had received feedback were asked, “Approximately how many minutes did you spend reviewing your feedback?” (response options in 5-minute ranges), “How many of your own responses did you replay?”, and “How many of the exemplar (highly rated peer) responses did you play?” (response options of 0, 1-2, 3-4, and ≥5). Residents responded to 4 additional items about the usefulness of each feedback component (scores, personal recordings, exemplar recordings, and learning points) using a 5-point scale with labels from not at all to extremely.

### Statistical Analysis

Data analysis was performed from July to December 2023. We averaged ratings across items and raters to create an overall rating of each response. We then averaged response ratings across all 7 vignettes at time 1 to create an overall time 1 score, and across all 6 vignettes at time 2 to create a time 2 score. We created a dichotomous disclosure exposure variable by combining disclosure involvement level and the number of times participated in disclosure.

To address our primary study question about the effect of the intervention (ie, access to VCA feedback), we conducted a factorial ANCOVA examining the impact that the intervention and prior disclosure exposure had on the primary outcome, time 2 scores, while adjusting for time 1 scores. We conducted a modified intention-to-treat analysis, including all residents with both time 1 and time 2 data. However, those who did not complete time 2 were necessarily excluded from analysis because they did not provide data for the main outcome. Post hoc tests examining the difference between the intervention and control group for each level of prior disclosure exposure were conducted using the Bonferroni correction. We used a Wilcoxon rank sum test to compare performance across specialties on overall scores. We used logistic regression to investigate whether time 1 scores were associated with the likelihood that participants returned for time 2. All statistical analysis was performed in R statistical software version 4.1.2 (R Project for Statistical Computing), with a 2-sided *P* < .05, except with ANCOVA, which is inherently 1-sided.

## Results

### Participant Characteristics

Programs identified 181 PGY2 residents available to attend educational conferences protected for VCA use (25 FM and 156 IM). Of these, 146 completed the VCA at time 1 before randomization (87 [60.0%] aged 25-29 years; 60 female [41.0%]; 77 male [53.0%]; 2 nonbinary [1.0%]) (Figure 1). Of the 146 residents randomized, 103 (70.5%) completed the VCA at time 2 (53 randomized to intervention, and 50 randomized to control). All responses of these 103 residents were rated by at least 6 raters. Of the 43 who only completed time 1, we omitted 10 whose responses were rated by 5 or fewer raters to ensure adequate reliability of scores. Table 1 shows participants’ demographic characteristics. We recruited 592 raters via MTurk. Of these, 187 (32.0%) were removed for providing poor-quality data consistent with inattentiveness, resulting in a final rater sample of 405 (eTable 2 in Supplement 2). After removing inattentive raters, each response was rated by 6 to 18 laypeople (mean [SD], 9.50 [1.60] individuals).

**Table 1.** Characteristics of Resident Physicians

Characteristic	Residents, No. (%)			
	Completed both time 1 and time 2 (n = 103)		Completed time 1 only (n = 33)^a^	All participants (N = 146)
	Intervention (feedback available before time 2) (n = 53)	Control (feedback not available until after time 2) (n = 50)		
Residency specialty				
Internal medicine	49 (92.5)	42 (84.0)	22 (66.7)	121 (83.0)
Family medicine	4 (7.5)	8 (16.0)	11 (33.3)	25 (17.0)
Age group, y				
25-29	34 (64.2)	26 (52.0)	23 (69.7)	87 (60.0)
30-34	14 (26.4)	19 (38.0)	8 (24.2)	44 (30.0)
≥35	4 (7.5)	4 (8.0)	2 (6.1)	11 (8.0)
Prefer not to say	1 (1.9)	1 (2.0)	0	4 (3.0)
Gender				
Female	22 (41.5)	17 (34.0)	17 (51.5)	60 (41.0)
Male	29 (54.7)	28 (56.0)	15 (45.5)	77 (53.0)
Nonbinary	0	1 (2.0)	1 (3.0)	2 (1.0)
Prefer not to say	2 (3.8)	4 (8.0)	0	7 (5.0)
Race				
African American or Black	2 (3.8)	1 (2.0)	0	3 (2.0)
Asian	11 (20.8)	12 (24.0)	7 (21.2)	31 (21.0)
Native Hawaiian or Pacific Islander	1 (1.9)	1 (2.0)	0	2 (1.0)
White	27 (50.9)	24 (48.0)	23 (69.7)	79 (54.0)
>1 Race	3 (5.7)	0	1 (3.0)	4 (3.0)
Other^b^	4 (7.5)	5 (10.0)	2 (6.1)	11 (8.0)
Prefer not to say	5 (9.4)	7 (14.0)	0	16 (11.0)
Hispanic or Latinx ethnicity	5 (9.4)	1 (2.0)	2 (6.1)	8 (5.0)
No. of times personally participated in error disclosure				
0	29 (54.7)	23 (46.0)	15 (45.5)	69 (47.0)
1	11 (20.8)	8 (16.0)	6 (18.2)	26 (18.0)
2-5	13 (24.5)	18 (36.0)	12 (36.4)	49 (34.0)
6-10	0	1 (2.0)	0	2 (1.0)
Highest level of involvement in error disclosure conversation				
Never participated	24 (45.3)	21 (42.0)	14 (42.4)	61 (42.0)
I observed silently	9 (17.0)	3 (6.0)	4 (12.1)	17 (12.0)
I spoke occasionally	11 (20.8)	8 (16.0)	5 (15.2)	30 (21.0)
I spoke frequently	0	6 (12.0)	2 (6.1)	9 (6.0)
I led the conversation	9 (17.0)	12 (24.0)	8 (24.2)	29 (20.0)

^a^ Ten participants who completed time 1 but not time 2 were omitted because of insufficient numbers of ratings.

^b^ Other was a choice that could be picked by the user by clicking the button next to that text if they felt that was the description of their race that was the best response.

The 53 participants in the intervention group completed surveys about interacting with the VCA feedback available before time 2. Two surveys lacked data because of electronic storage errors. Of the 51 residents with survey data, 28 (54.9%) reported that they had reviewed their feedback before the survey, reporting variable total periods of time in review; 7 (13.7%) spent less than 5 minutes, 12 (23.5%) spent 6 to 10 minutes, 5 (9.8%) spent 11 to 16 minutes, 3 (5.9%) spent 16 to 20 minutes, and 1 (2.0%) spent 21 to 25 minutes in review. Residents reported listening to variable numbers of their own or exemplar responses, but reported listening to more exemplar responses (Table 2). Residents rated the usefulness of the 4 feedback components similarly (Table 2).

**Table 2.** Survey Responses of Resident Physicians Randomized to Receive Crowdsourced Feedback About Error Disclosure Skills

Survey question and response options	Residents responding, No. (%) (n = 51)^a^
How many of your own responses did you replay?	
0	29 (56.8)
1-2	15 (29.4)
3-4	5 (9.8)
≥5	2 (3.9)
How many of the exemplar (highly rated peer) responses did you play?	
0	27 (52.9)
1-2	11 (21.6)
3-4	10 (19.6)
≥5	3 (5.9)
How useful was seeing how your response compared to other responses?	
Extremely	2 (3.9)
Very	7 (13.7)
Somewhat	10 (19.6)
A little	7 (13.7)
Not at all	1 (2.0)
Did not use	24 (47.0)
How useful was seeing the learning points for each vignette?	
Extremely	2 (3.9)
Very	10 (19.6)
Somewhat	9 (17.6)
A little	8 (15.7)
Not at all	0
Did not use	22 (43.1)
How useful was being able to listen to your own response for each vignette?	
Extremely	2 (3.9)
Very	7 (13.7)
Somewhat	10 (19.6)
A little	7 (13.7)
Not at all	1 (2.0)
Did not use	24 (47.0)
How useful was being able to listen to highly rated responses for each vignette?	
Extremely	2 (3.9)
Very	8 (15.7)
Somewhat	8 (15.7)
A little	5 (9.8)
Not at all	1 (2.0)
Did not use	27 (52.9)

^a^ Responses sum to 51 rather than 53 because a data transfer issue caused data loss from 2 completed surveys. Those who did not report reviewing feedback at the time of the survey could have reviewed it at a later time before time 2.

### Communication Rating Outcomes

**Figure 3.**
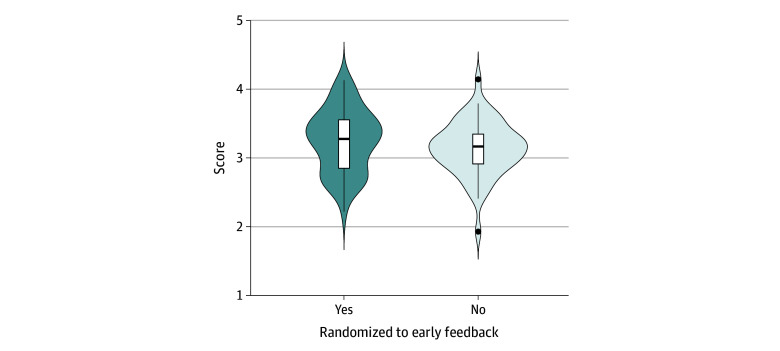
Violin Plot of Participants’ Video-Based Communication Assessment Scores by Study Group The vertical line represents the median, the white bar represents the IQR, the horizontal line represents 1.5 times the IQR, and the dots represent outliers. The curve represents an estimation of the distribution shape of the data.

Figure 3 displays the distribution of crowdsourced ratings by intervention assignment (eTable 3 in Supplement 2 presents time 1 ratings). High performers were rated 2 points higher than low performers on a 5-point scale. The ANCOVA model, which included time 1 scores as a covariate, showed a significant main effect of the intervention; the mean (SD) time 2 overall scores were 3.26 (0.45) for the intervention group and 3.14 (0.39) for the control group (difference, 0.12; 95% CI, 0.08-0.48; η_p_^2^ = 0.04; *P* = .01). We also detected a significant interaction between the intervention (ie, feedback availability) and prior exposure to disclosure conversation (η_p_^2^ = 0.05; *P* = .03) after adjusting for time 1 scores (eTable 4 in Supplement 2). Post hoc comparisons using Bonferroni correction revealed that when residents had no prior disclosure exposure, those in the feedback intervention group scored significantly higher than those in the control group (mean [SD] score, 3.33 [0.43] vs 3.09 [0.44]; difference, 0.24; 95% CI, 0.01-0.48; *P* = .007) at time 2.

We did not observe a significant difference in communication skill performance between IM and FM residents (mean [SD] score, 3.24 [0.44] vs 3.26 [0.27]). Logistic regression found a significant association between time 1 scores and the likelihood that a participant returned for time 2, such that a 1-unit increase in time 1 scores corresponded to a 2.89-fold increase in the odds of participants completing time 2 (odds ratio, 2.89; 95% CI, 1.06-7.84; *P* = .04).

## Discussion

This multisite, randomized clinical trial found that using VCA to provide crowdsourced feedback to PGY2 IM and FM residents about error disclosure skills was associated with an improvement in these skills. Feedback was most impactful among residents who reported they had not been exposed to error disclosure in clinical care, suggesting this intervention could be particularly beneficial at an earlier phase of training. Our findings highlight the potential for the VCA as a scalable practice tool for training that would be logistically challenging to replicate with standardized patients.

Despite these encouraging findings, surveys revealed that many residents either did not review or spent minimal time reviewing their feedback, which likely blunted the intervention’s effect. To optimize the VCA’s efficacy, future research should investigate and resolve barriers to residents’ use of crowdsourced feedback. Possible barriers in this trial included the delay between practice and feedback, the lack of protected time to review feedback, a need for adjunctive coaching, unidentified shortcomings of the feedback content and presentation, or the need for more practice repetitions. If confirmed, some of these potential barriers can be addressed with technical or curricular changes, such as providing dedicated time for feedback review or a paired faculty coach. However, using crowdsourcing to incorporate the layperson’s voice in statistically reliable feedback currently requires at least 2 to 3 days, making it difficult to provide instantaneous results.

To our knowledge, this study represents the largest assessment of medical error communication skills among IM and FM residents using a standardized instrument. Although all participants received a lecture on practical error disclosure skills, we observed significant variation in their performance, with high performers rated 2 points higher than low performers on a 5-point scale. Self-reported disclosure exposure did not explain this variation. These findings suggest that common teaching approaches leave at least a subset of residents unprepared for effective error disclosure and affirms the Accreditation Council for Graduate Medical Education’s requirement that residents practice these skills.

The reliability of VCA scores for comparative assessment may be useful for residency directors evaluating milestone progress within their programs. Yet, the VCA was intended for formative use by individuals and should first be optimized for uses that residents find engaging and psychologically safe. Of particular concern, we found that worse performance at time 1 was associated with not completing time 2. One possible explanation is that participants who found the exercise difficult left discouraged. The departure of individuals with the most room for improvement highlights a difficulty for educators and health system leaders tasked with preparing all physicians for effective error disclosure. Future work should determine approaches that better engage low performers in deliberate practice, including repetition and coaching.

### Limitations

Our work has limitations. First, statistical power was reduced by both nonparticipation with the intervention and dropout before the second VCA use. Second, survey results may be affected by social desirability and recall bias. Third, there is no established score benchmark for competence or mastery, limiting contextualization of the observed effect size. Fourth, we relied on self-report of feedback review, rather than direct measurement; the software does not currently track time spent in feedback activities. Because of the timing of survey administration, residents who reported not reviewing feedback could have theoretically chosen to delay taking the VCA to review feedback instead. However, if this had occurred, it would have diminished, not increased, the effect size. Fifth, reviewing layperson responses to remove those with low contribution to reliability requires effort that may not scale to very widespread use. Sixth, the crowdsourced laypeople were predominantly White and non-Hispanic; lack of racial diversity in the rating pool may introduce unmeasured bias in the results. Seventh, unmeasured confounders missing from analysis may have affected the results. The study has important strengths, including a large geographically diverse cohort with robust participation, suggesting the findings may generalize to other IM and FM residencies.

## Conclusions

In summary, this study found that self-directed review of crowdsourced feedback was associated with error disclosure skill improvement in IM and FM residents who had already received a lecture on the topic. The VCA has the potential to solve a widely unmet need for graduate medical education patient safety educators. Future work should determine the viewpoints of residency leaders and residents about how the tool can be improved for curricular adoption, and eventually to evaluate its effect on patient-reported communication outcomes.

## Notes

Supplement 1.
Trial Protocol and Statistical Analysis Plan

Supplement 2. **eTable 1.** Video communication assessment cases used in a randomized controlled trial of an intervention designed to improve resident adverse event communication skills **eTable 2.** Demographics of laypeople crowdsourced via Amazon Mechanical Turk (MTurk) who provided attentive ratings of resident adverse event communication skills **eTable 3.** VCA ratings at Time 1 (baseline) **eTable 4.** ANCOVA table for the impact of feedback and disclosure exposure on time 2 scores

Supplement 3.
Data Sharing Statement
