# Supplementary material for: Crowdsourced Feedback to Improve Resident Physician Error Disclosure Skills: A Randomized Clinical Trial
Source: JAMA Netw Open. 2024 Aug 7;7(8):e2425923. doi: 10.1001/jamanetworkopen.2024.25923 (PMC11307134; doi:10.1001/jamanetworkopen.2024.25923)
Supplement: Supplement 1. — Trial Protocol and Statistical Analysis Plan [file jamanetwopen-e2425923-s001.pdf]

1. Study Purpose and Research Objectives

The Video-based Communication Assessment (VCA) is a novel tool created by the NBME to support physicians seeking to improve their communication skills through practice and feedback. While initial feasibility and reliability studies yielded promising results, no studies have yet examined whether VCA feedback is effective in improving physicians' communication skills. A high priority for graduate educators is: Does the VCA improve resident physicians' error disclosure skills? We will conduct a pre-post randomized trial of the VCA with 100 resident subjects. We will also enroll health system leaders and residency program leaders to answer four total research questions:

1) What is the effectiveness of VCA feedback for resident error disclosure skill development?

2) Does the VCA discriminate between inexperienced physicians and those with extensive error disclosure experience?

3) How can VCA feedback be optimized for resident learners?

4) What makes VCA implementation acceptable, appropriate, and feasible for graduate medical education (GME) leaders?

2. Background and Rationale

Poor communication after a medical injury often leaves patients and families feeling alone, afraid, confused, abandoned, and more likely to seek information through malpractice claims.[1,2] To meet patient needs for transparency and support, as well as ACGME mandates, resident physicians require training for responding to events ranging from common minor complications to rare, harmful errors.[3] Available techniques for teaching communication skills, such as lectures and standardized patient scenarios, have critical limitations.[4] Residents also learn through clinical practice, but opportunities to practice high-stakes disclosures are infrequent and seldom accompanied by formative feedback from supervising physicians.[5] New approaches and incorporation of the patient voice are sorely needed. The Video-based Communication Assessment (VCA) has emerged to meet this need, and preliminary research has established that crowdsourced laypeople generate actionable, reliable feedback.[6,7] Whether use of the VCA is associated with improved communication skills is not known.

We recently created proof-of-concept VCA cases of harmful medical errors for 3 specialties (internal medicine, pathology, and obstetrics). We enrolled residents at the University of Washington (UW) as respondents and determined the number of raters needed to achieve reliable scores. Early analysis also indicates that the VCA can detect differences between residents in their ability to meet patient needs in simulated harm situations. However, this study was not embedded in a curriculum, meaning it did not measure the effect of VCA alongside standard error disclosure instruction through combined lecture and practice. Additionally, the sample size was too small to draw conclusions about educational outcomes. To build on these promising

early findings, it is critical to study the VCA's effectiveness for promoting skill acquisition in the context of real-world resident curricula with an adequately powered trial.

Before adopting the VCA as an educational tool, GME leaders will seek evidence of its effectiveness *and* direction about how to best implement the instrument within a communication curriculum. A well-designed VCA trial offers an important opportunity to study questions of importance to residency leaders that can inform how the NBME offers the VCA as a product. For example, residency leaders may want information on mastery performance to contextualize results shared with residents. Unfortunately, this instrument has not been tested among senior leaders with extensive experience with error disclosure. Additionally, educators may ask for guidance on curricular integration and information on the burden of implementation. To meet this need, we will investigate how to optimize the usefulness of VCA feedback and makes long-term curricular implementation feasible and practical for educators. This information will be important for the NBME as it makes plans to offer the NBME more widely.

3. Aims, methods, and analysis

Overview:

We will investigate the effectiveness of VCA feedback through a multi-site randomized trial. Internal medicine and family medicine residency programs affiliated with UW (WA and ID), Washington State U. (WA), Dartmouth (NH), U. of Massachusetts (MA), Wash U in St. Louis (MO), and Beaumont Health (MI) will conduct pre/post VCA assessments as part of an adverse event communication curriculum during the R2 year. Residents will be randomized to immediate or delayed feedback to determine the contribution of personalized feedback and examples of excellent disclosure on their communication skills ratings at the second use of VCA. Alongside this educational intervention, we will a) measure the VCA performance of senior health professionals with extensive error-disclosure experience and b) use surveys and semi-structured interviews to understand how faculty and residents prefer to receive and apply the VCA feedback. Existing and new VCA cases with greater variety will be used to assess resident adverse event communication skills at two timepoints per cohort: baseline and 3 weeks.

Aim 1: What is the effectiveness of VCA feedback for individual error disclosure skill development?

<u>Participants</u>: A cohort of 100 second year residents in internal medicine and family medicine. This sample size (50 in each trial arm) will provide 85% power to detect a modest effect size of 0.15 on a 5-point scale, based on average score of 3.53 observed in pilot studies.

<u>Interventions</u>: All residents will receive mandatory classroom-based training after the first assessment, consisting of lecture and role-play training on disclosing serious harmful errors (figure 1). Incentives would not be provided as this would be part of required education.

<u>Data</u>: Resident responses to each vignette will be rated by panels of 10 crowdsourced laypeople on 6 items with a 5-point scale, addressing the following domains: (being accountability for their actions, being honest about what happened, being sincerely sorry, understanding how the patient was feeling, caring for the patient, and overall response). The summary score generated from combining these ratings was validated in pilot studies as a reliable measure of resident error disclosure skill. We will measure residents' summary communication scores for Set A (time 0) and Set B (3 weeks) (see figure below).

<u>Analysis</u>: The trial will compare Set B communication scores for residents who received the feedback intervention *before* the second VCA versus those who received it *after* the second VCA using an ANCOVAR with Set A as a covariate and Set B scores as the dependent measure. The analysis would determine the effect of feedback, taking into account any pre-existing differences on Set A, including demographic responses to in-app surveys.

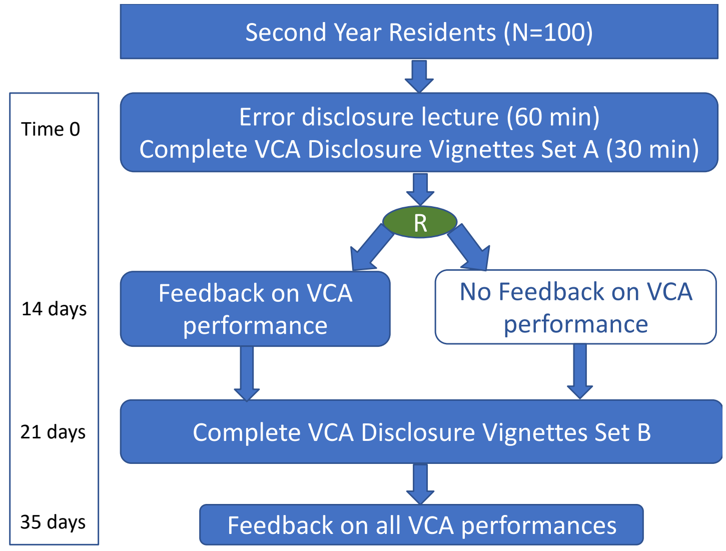

Figure 1: Resident VCA randomized trial design

Aim 2: How are VCA responses from healthcare leaders with extensive error disclosure experience rated?

<u>Participants</u>: A cohort of 12 individuals with experience in error disclosure and a reputation for excellent communication skills. These individuals will be recruited through the Collaborative for Accountability and Improvement (CAI) and offered an incentive for participation. The CAI is a national network of leaders, researchers and advocates collaborating to improve how healthcare institutions communicate and respond after patients are harmed. To qualify for the study, participants must self-report involvement in at least 10 disclosures of serious harm. Typical roles held by these people might be medical director or risk manager.

Data: The same crowdsourced communication rating used in aim 1 will be applied. Participants will respond to all of the VCA cases used by residents in Set A and B.
Analysis: T-tests of average scores will be compared with those generated by residents.

Aim 3: How would residents improve the effectiveness of formative feedback from the VCA?
Participants: We will recruit 20 residents who have been through the VCA trial in Aim 1 for two focus groups to determine the properties of the VCA they believe catalyze behavior change.
Data: Focus group questions will include an inquiry about overall user experience, which elements of feedback were most likely to support behavior change, how comments from raters could be more informative, and what is the ideal role of faculty as an ally or coach in responding to feedback.
Analysis: Standard qualitative methods will be applied, using content analysis of transcripts to distill findings.

Aim 4: What makes VCA implementation acceptable, appropriate, and feasible for educators?
Participants: 10 residency leaders (program directors, assoc. program directors, and asst. program directors) at the participating sites. Participation incentives will be offered.
Data: Mixed methods will be used due to the small sample of participating educators. Implementation surveys using validated scales[8] will assess the acceptability, appropriateness and feasibility of incorporating VCA into a residency curriculum. Participants will also join a focus group to discuss the VCA implementation. Focus group questions will include an inquiry about overall experience, VCA features that facilitated or obstructed adoption, and recommendations for future software and content development.

References

1. Delbanco T, Bell SK. Guilty, afraid, and alone – struggling with medical error. NEJM. 2007: 357 (17); 1682-3.

2. Gallagher TH, Studdert D, Levinson W. Disclosing Harmful Medical Errors to Patients. NEJM, June 2007:356 (26); 2713-9

3. White AA, Bell SK, Krauss MJ, et al. How Trainees Would Disclose Medical Errors: Educational Implications for Training Programs. Med Educ, April 2011: 45 (4); 372-380.

4. Stroud L, Wong BM, Wollenburg E., et al. Teaching Medical Error Disclosure to Physicians-in-training: A Scoping Review. Acad Med, 2013: 88 (6); 884-892

5. White AA, Gallagher TH, Krauss MJ, et al. The attitudes and experiences of trainees regarding disclosing medical errors to patients. Acad Med, March 2008: 83 (3); 250-256

6. Mazor KM, King AM, Hoppe RB, et al. Video-Based Communication Assessment: Development of an Innovative System for Assessing Clinician-Patient Communication. JMIR Med Educ. 2019 Feb 14;5(1):e10400.

7. Mazor KM, King AM, Hoppe RB, et al. Using crowdsourced analog patients to provide feedback on physician communication skills. Patient Educ Couns. 2021 Mar;S0738-3991(21)00169-5.

8. Weiner BJ, Lewis CC, Stanick C, et al. Psychometric assessment of three newly developed implementation outcomes measures. Implementation Science. 2017: 108 (12).
