# Supplementary material for: Crowdsourced Feedback to Improve Resident Physician Error Disclosure Skills: A Randomized Clinical Trial
Source: JAMA Netw Open. 2024 Aug 7;7(8):e2425923. doi: 10.1001/jamanetworkopen.2024.25923 (PMC11307134; doi:10.1001/jamanetworkopen.2024.25923)
Supplement: Supplement 2. — eTable 1. Video communication assessment cases used in a randomized controlled trial of an intervention designed to improve resident adverse event communication skills eTable 2. Demographics of laypeople crowdsourced via Amazon Mechanical Turk (MTurk) who provided attentive ratings of resident adverse event communication skills eTable 3. VCA ratings at Time 1 (baseline) eTable 4. ANCOVA table for the impact of feedback and disclosure exposure on time 2 scores [file jamanetwopen-e2425923-s002.pdf]

Supplemental Online Content

White AA, King AM, D'Addario AE, et al. Crowdsourced feedback to improve resident physician error disclosure skills: a randomized clinical trial. *JAMA Netw Open*. 2024;7(8):e2425923. doi:10.1001/jamanetworkopen.2024.25923

eTable 1. Video Communication Assessment cases used in a randomized controlled trial of an intervention designed to improve resident adverse event communication skills

eTable 2. Demographics of laypeople crowdsourced via Amazon Mechanical Turk (MTurk) who provided attentive ratings of resident adverse event communication skills

eTable 3. VCA ratings at Time 1 (baseline)

eTable 4. ANCOVA table for the impact of feedback and disclosure exposure on time 2 scores

This supplemental material has been provided by the authors to give readers additional information about their work.

© 2024 White AA et al. *JAMA Network Open.*

eTable 1. Video Communication Assessment cases used in a randomized controlled trial of an intervention designed to improve resident adverse event communication skills

**Cases at Time 1**			
Vignette	Situation description (to physician)	Situation Description (to rater)	What the patient says
1A	A 45-year-old male is admitted to orthopedics ten days ago for a left hip fracture. He was transferred to you with postoperative hyponatremia from SIADH and mental status changes. Postoperative left leg swelling was noted and a duplex scan found a deep venous thrombosis. The patient was started on enoxaparin at the standard dose of 1 mg/kg every 12 hours with daily weights. Four days later, the patient has a massive upper GI bleed requiring six units of blood. An endoscopy finds diffuse gastritis.<br>On reviewing the chart, you notice that the patient's admission weight is listed as 160 kg, which seems high for the size of the patient. No weights had been recorded since. You ask for the patient to be re-weighed, and his weight is 85 kg, not 160 kg. Based on this faulty admission weight, the patient has been receiving nearly twice as much enoxaparin as he needed, which has led to this GI bleed. The nurse has told him his blood was too thin and he's asked you to explain further. You are now meeting to disclose the error.	Dan Iglesias was admitted to the hospital ten days ago for a hip fracture and developed a blood clot in his leg. He was placed on a blood thinner, but developed serious bleeding. The nurse told him his blood was too thin, and that the doctor needed to explain more.<br>The doctor sat down to discuss the situation and the patient says:	"I heard I bled because my blood was too thin, but I haven't had that problem before. Can you explain what is going on?"

1B	You've explained to the patient that he suffered bleeding because an error was made in measuring his weight. However, he remains unclear on who is responsible. As you look at the patient, it is visibly obvious he does not weigh 160 Kg. He asks:	Mr. Iglesias understands that the wrong weight was in his medical chart and it caused him to get too much blood thinner. He asks:	"So you mean all of this bleeding could have been avoided? …whose fault is this?"
1C	You've explained that several members of the care team misjudged his weight and none, including yourself, questioned the details in the chart. He asks:	Mr. Iglesias understands that several members of his care team missed chances to recognize and fix the wrong weight in the chart. He asks:	"Do you have any idea how terrible this has been for me?"
1D	You have expressed empathy for his unnecessary suffering. He is coming to terms with the error, but has further questions. He asks:	The doctor has shared information about the mistake, but Dan asks:	"I'm glad you're leveling with me about what happened, but how am I supposed to trust you now?"
2A	Marvin Williams is your primary care patient. He is 82 years old and frail due to heart failure, diabetes, and poor mobility. You last saw him 2 days ago via video visit.<br>During your video visit he complained of jaw pain, dizziness, and malaise for several days. He shared his home BP log, which had several systolic readings in the 90's. You were concerned about temporal arteritis and asked him to get labs and start 40 mg of prednisone daily. His WBC was 15.1 k/mL with a left shift and ESR was normal, making temporal arteritis less likely. You saw the results and ask your staff to have him come in the next day to re-evaluate. They leave a message on his voicemail. You curbside a colleague, who agrees with your plan.<br>The next day you learn he was admitted with staph bacteremia	Marvin Williams is 82 years old and has poor health. He saw his doctor 2 days ago in a video visit. He was dizzy, tired, and his jaw hurt for several days. He shared his home blood pressure record with his doctor, with several low readings.<br>The doctor said Mr. Williams' immune system was attacking an artery near the jaw. The doctor ordered lab tests and prescribed a medicine to reduce inflammation. Mr. Williams went to the lab; the clinic left a voicemail to come in for an in-person visit.<br>He felt worse that night and was rushed to the hospital. He was treated in intensive care for a different diagnosis: a bloodstream	"Argh. Not too good. I feel like I've been run over by a truck. This has been one of the toughest things I've gone through. I guess I should've called you sooner. What did I do wrong?"

© 2024 White AA et al. *JAMA Network Open.*

	requiring pressors. No clear source was found, but he has stabilized on antibiotics and remains on some steroids for sepsis. You stop by the hospital to see him and ask how he is feeling now.	infection. His condition is stable now, but he nearly died. His doctor stops by to check on him and asks him how he is feeling.	
2B	You've shared information about the way you formulated a diagnosis, including that the labs and the jaw pain pointed to different diagnoses.	The doctor shares that the lab tests and jaw pain suggested different reasons for his illness. Mr. Williams replies:	"…so you had a lab test that said I was pretty sick? You didn't tell me that I could get this sick. How can I be sure this won't happen to someone else?"
2C	You agree that catching this sooner and recommending going to the ER immediately would have reduced the severity of his illness.	The doctor agrees that catching this sooner might have helped. Mr. Williams nods and says:	"My wife used to call me a stubborn old mule. Still, I did what she asked and got in to see the doctor. I'm overwhelmed, and don't really know how we're going to pay for this…"
**Cases at Time 2**			
Vignette	Situation description (to physician)	Situation Description (to rater)	What the patient says
3A	You are a primary care doctor for a 48-year-old woman with diabetes. Today she mentions a breast lump that is new to her. You review her chart and see a mammogram report from last year had a suspicious	Lorna Smith visits her primary care doctor to evaluate a new breast lump. She figures it isn't anything serious because she had a mammogram last year and	"When I didn't hear from your office about the mammogram, I assumed everything was normal. Was there any sign of this

© 2024 White AA et al. *JAMA Network Open.*

	calcification with recommendation for biopsy. At her last health maintenance visit you did not document a plan for the result and do not recall seeing it before now. You examine her and the site of the lump corresponds to the location on x-ray. She says:	never heard about any abnormal results. The doctor examined her and she changed back to regular clothes. She wants to discuss the lump now and says:	lump on the test last year?”
3B	You've told the patient that there were early warning signs of possible breast cancer on her mammogram one year ago. She says:	Ms. Smith has learned that her mammogram last year showed early signs of possible breast cancer, but nothing was done about it. She is feeling panicked and says:	“This is terrible! I’ve never been more frightened…plus you’re telling me that we might have known about it a long time ago!”
3C	You've acknowledged how upsetting the error is. The patient now understands that there were early warning signs of possible breast cancer on her mammogram. She says:	Ms. Smith feels like the clinic and her doctor have failed her. She asks:	“How could this happen to me? I feel like I can’t trust anyone anymore. How am I supposed to believe your advice in the future?”
4A	Yvonne Davis is a 79 yo woman at a nursing home, recovering from a stroke. Her orders include physical therapy, protein shakes, and regular repositioning. She intermittently declines these interventions, telling the nurse that she ‘doesn’t feel like it'. She’s struggled with urinary incontinence, and her daughter requested a catheter. You have avoided ordering a catheter due to the infectious risk.<br>You normally round on your nursing home patients weekly, but were not able to do so last week due to an overbooked clinic. Today you learn that Ms. Davis has developed a pressure wound of significant depth. On review of the chart, the staff documented erythema on the sacrum last week, but didn’t contact you.	Yvonne Davis is at a nursing home for recovery from a stroke. Her orders include treatments to reduce pressure sores, like exercises and nurses turning her in bed regularly. However, she often declines, telling the nurse that she ‘doesn’t feel like it'. She also has urine incontinence. Her daughter, Tamika Ellis, asked the doctor for a bladder catheter to keep her bottom dry. The doctor avoided this because catheters can cause urinary infections, but the skin on her backside becomes fragile.<br>The doctor normally visits weekly, but didn’t come last week. Today the	“Doctor, this is exactly what I was worried about! My mother now has a sore the size of my fist on her tailbone! What a disaster! How did this happen?”

© 2024 White AA et al. *JAMA Network Open.*

	When you approach her room, you see her daughter, Tamika Ellis, whom you have met previously. After you greet her, Ms. Ellis says:	nurses found a pressure sore on Ms. Davis' tailbone that hurts and will take weeks to heal.<br><br>The doctor comes to visit and first talks with her daughter, Ms. Ellis, in the hallway.	
4B	You tell Ms. Ellis that you are very sorry that her mother has developed this sore, and begin to explain what may have contributed. Ms. Ellis responds:	The doctor discusses some factors leading to the pressure sore. Ms. Ellis responds:	"I may not be a doctor, but I'd bet my bottom dollar that my mother wouldn't have this if she'd had a catheter like I said she needed. Is there going to be an investigation?"
4C	You acknowledge that a catheter would have kept her skin dry. Ms. Ellis replies:	The doctor discusses the catheter. Ms. Ellis replies:	"Somebody's got to keep a better eye on these nurses, and make SURE that they move her and keep her dry. I don't get the sense that you or anyone else is taking this seriously."

© 2024 White AA et al. *JAMA Network Open.*

eTable 2: Demographics of laypeople crowdsourced via Amazon Mechanical Turk (MTurk) who provided attentive ratings of resident adverse event communication skills

Characteristic	N	%
**Age**		
20-24	12	2.96
25-29	50	12.35
30-34	76	18.77
35-39	81	20.00
40-44	63	15.56
45-49	37	9.14
50-54	29	7.16
55-59	29	7.16
60-64	13	3.21
65+	15	3.70
**Gender**		
Female	182	44.94
Male	221	54.57
Non-binary	1	0.25
Prefer not to say	1	0.25
**Race**		
American Indian or Alaska Native	5	1.23
Asian	20	4.94
Black or African American	26	6.42
White	329	81.23
More than one race	17	4.20

© 2024 White AA et al. *JAMA Network Open.*

Other	6	1.48
Prefer not to say	2	0.49
**Hispanic Ethnicity**		
Yes	28	6.91
No	374	92.35
Prefer not to say	3	0.74
**Education**		
Some high school	4	0.99
High school or equivalent	69	17.04
Some college, no degree	68	16.79
Associate degree	44	10.86
Bachelor’s degree	183	45.19
Graduate degree	37	9.14

© 2024 White AA et al. *JAMA Network Open.*

eTable 3. VCA ratings at Time 1 (baseline)

For those who completed both rounds (N = 103) the mean time 1 score was 3.28 (SD = .37). For those later assigned to the intervention group the mean score was 3.25 (SD = .35) and it was 3.32 (SD = .35) for those later assigned to the control group.

eTable 4: ANOVA table for the impact of feedback and disclosure exposure on time 2 scores

Source	df	SS	F	p
Feedback	1	1.08	7.51	0.01
Disclosure Exposure	1	0.11	0.76	0.38
Feedback : Disclosure Exposure	1	0.70	4.83	0.03
Residuals	98	14.97		

© 2024 White AA et al. *JAMA Network Open.*
