# Supplementary material for: Crowdsourced Feedback to Improve Resident Physician Error Disclosure Skills: A Randomized Clinical Trial
Source: JAMA Netw Open. 2024 Aug 7;7(8):e2425923. doi: 10.1001/jamanetworkopen.2024.25923 (PMC11307134; doi:10.1001/jamanetworkopen.2024.25923)
Supplement: Supplement 3. — Data Sharing Statement [file jamanetwopen-e2425923-s003.pdf]

Data Sharing Statement

White. Crowdsourced Feedback to Improve Resident Physician Error Disclosure Skills. *JAMA Netw Open*. Published August 07, 2024. doi:10.1001/jamanetworkopen.2024.25923

Data
Data available: No

Additional Information
Explanation for why data not available: The original data consists of audio recordings of individual physicians that are potentially identifiable if released. In addition, the participants did not consent to have their recordings released to other researchers.
